# Development of novel nomograms for predicting prostate cancer in biopsy-naive patients with PSA < 10 ng/ml and PI-RADS ≤ 3 lesions

**DOI:** 10.3389/fonc.2024.1500010

**Published:** 2025-01-07

**Authors:** Jia-gui Chai, Yu-hang Li, Chang-xing Ke

**Affiliations:** Department of Urology, The Second Affiliated Hospital of Kunming Medical University, Kunming, China

**Keywords:** prostate cancer, PI-RADS, prostate-specific antigen, nomogram, diagnosis

## Abstract

**Purpose:**

To develop novel nomograms for predicting prostate cancer (PCa) and clinically significant prostate cancer (csPCa) in patients with prostate-specific antigen (PSA) < 10 ng/ml and PI-RADS v2.1 score ≤ 3.

**Methods:**

We retrospectively collected data from 327 men with PSA < 10 ng/ml and PI-RADS score ≤ 3 from June 2020 to June 2024 in our hospital. Clinical data were compared among the PI-RADS scores 1-3 population, PI-RADS scores 1-2 population, and PI-RADS score 3 population. Logistic regression analyses were conducted to identify independent risk factors for PCa or csPCa, and nomograms were subsequently developed. The nomograms were evaluated via receiver operating curves (ROC), calibration curves, and decision curve analysis (DCA). Internal validation was conducted using bootstrap methods.

**Results:**

Among the 327 patients, 224 (68.50%) were diagnosed with benign, 65 (19.87%) with csPCa, and 38 (11.62%) with clinically insignificant prostate cancer (cisPCa). Prostate-specific antigen density (PSAD), lesion volume (LV), lesion location, and apparent diffusion coefficient (ADC) were found to be independent risk factors for PCa and csPCa in PI-RADS scores 1-3 population. PSAD and lesion location were independent risk factors for PCa in the PI-RADS scores 1-2 population, while PSAD, lesion location and ADC were independent risk factors for PCa in the PI-RADS score 3 population. Four nomograms were established based on these variables. For the population with PI-RADS scores 1-3, the area under the ROC (AUC) for predicting PCa and csPCa was 0.78 and 0.79, respectively. For patients with PI-RADS scores 1-2, the AUC for predicting PCa was 0.75. For patients with PI-RADS score 3, the AUC for predicting PCa was 0.78. The calibration curves revealed good concordance between the predicted probability and the actual probability. DCA demonstrated the net benefit of nomograms. Internal validation revealed strong discrimination of the nomograms.

**Conclusion:**

We developed novel nomograms with acceptable discriminability for predicting PCa and csPCa in patients with PSA < 10 ng/ml and PI-RADS score ≤ 3. These models can assist urologists in determining the necessity of prostate biopsy.

## Introduction

Prostate cancer (PCa) is the second most common malignant tumor in men, with over 1.462 million new cases and 394,200 deaths worldwide each year (1). Increasing evidence indicates that early screening for PCa significantly reduces morbidity and mortality (2, 3). Currently, early PCa screening predominantly relies on multiparametric magnetic resonance imaging (mp-MRI) and prostate-specific antigen (PSA) testing (4, 5).

Based on mp-MRI, Prostate Image-Reporting and Data System (PI-RADS) score can be obtained, which provides clues for early PCa screening (6, 7). PI-RADS score ≤ 3 is generally classified as low-risk, often leading to prostate biopsy (PBx) not being recommended (8, 9). Additionally, PSA is widely used for early PCa screening (10). PSA < 10 ng/ml is considered a gray zone, leading to ongoing debate about the necessity of biopsy in these cases (11).

Recent studies have reported whether individuals with PI-RADS score ≤ 3 should undergo PBx (11, 12). However, decision-making becomes challenging in patients with PI-RADS scores 1-3 and PSA < 10 ng/ml, especially for patients with PI-RADS scores 1-2. To our knowledge, this issue is rarely explored. In this study, we aimed to address this clinical challenge by developing novel nomograms to predict the likelihood of PCa and clinically significant prostate cancer (csPCa) in patients with PI-RADS scores 1-3 and PSA < 10 ng/ml.

## Materials and methods

### Patients

This retrospective analysis was approved by the Institutional Review Board of our hospital. Patient informed consent requirement was waived. We evaluated clinical data from 1,808 patients who underwent mp-MRI and PBx at the Second Affiliated Hospital of Kunming Medical University from June 2020 to June 2024. The PBx criteria were: suspicious nodules detected by digital rectal examination, suspicious lesions detected by ultrasound or mp-MRI, PSA greater than 10 ng/ml, PSA at 4-10 ng/ml with abnormal f/t PSA and/or prostate-specific antigen density (PSAD). When one or more of the above were present, PBx was recommended (2). All patients received ultrasound-guided transrectal or transperineal biopsies, including 12 + x (12 cores in peripheral zone [PZ] and transitional zone [TZ]; x represents cores obtained from suspicious or positive areas by mp-MRI). The csPCa was defined as gleason scores (GS) ≥ 7, or > 3 biopsy cores positive, or at least one biopsy core with > 50% involvement. Clinically insignificant prostate cancer (cisPCa) was defined as GS < 7 without gleason pattern 4 or 5, less than 3 core samples, and no core sample > 50% involved (13). Biopsy-naive was defined as undergoing PBx for the first time and had not previously undergone any PBx or prostate surgery. The end point of the study was to obtain accurate and detailed pathological results for the biopsy-naive patient. Biopsy negative patients were regularly followed up (3 months/time).

**Figure 1:**
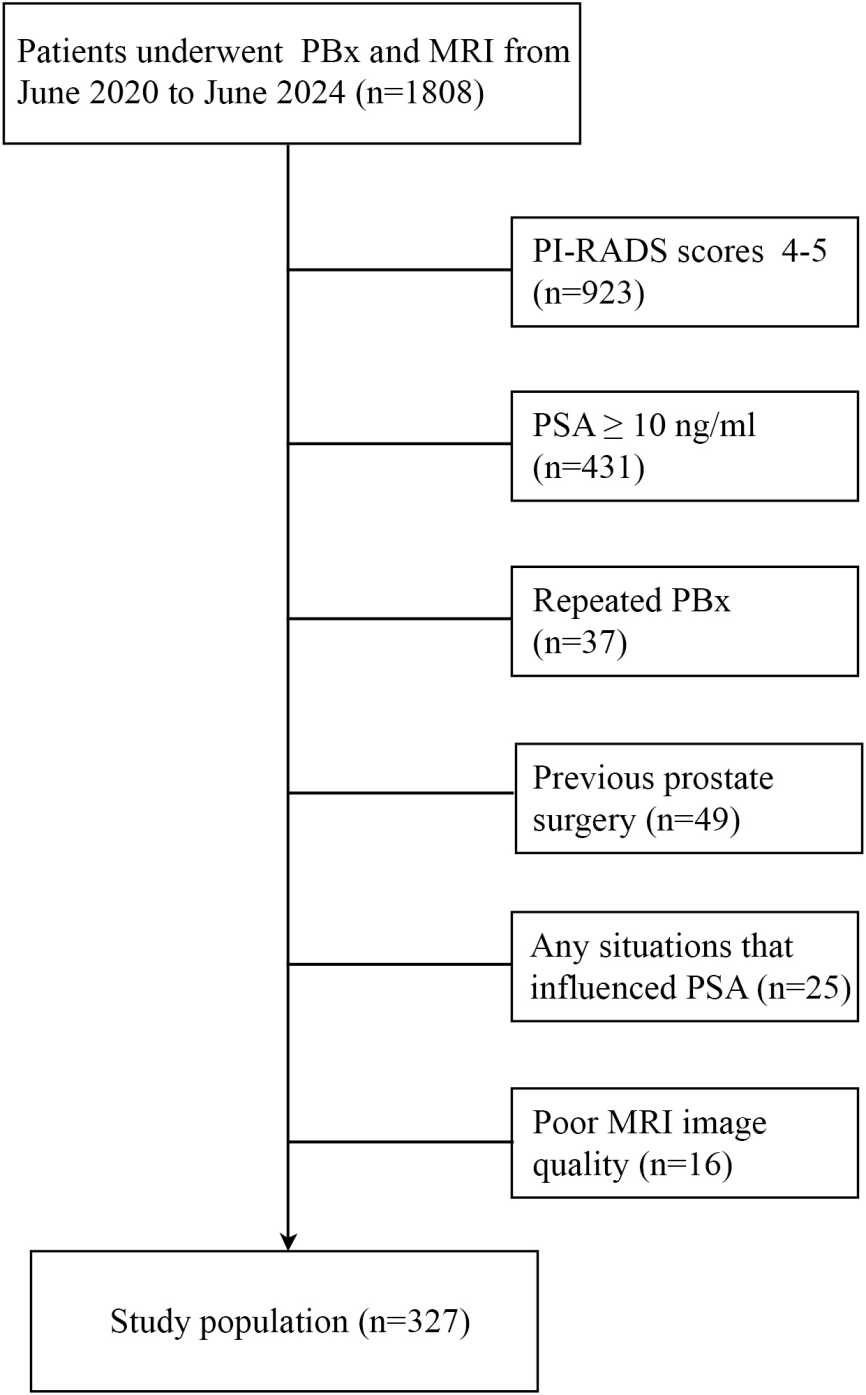
The detailed patient inclusion and exclusion follow charts.

The exclusion criteria included: (1) Any possible situations that influenced PSA, such as indwelling catheters. (2) poor mp-MRI image quality (the lesion volume and PI-RADS v2.1 score cannot be obtained through MRI). (3) repeated biopsy. (4) previous prostate surgery. (5) PI-RADS scores 4-5 or PSA ≥ 10 ng/ml. Ultimately, 327 men with PSA < 10 ng/ml and PI-RADS v2.1 score ≤ 3 were included in the study (**Figure 1**).

### MRI protocol

The prostate mp-MRI (Philips Achieva, Netherlands) was performed with a 3-Tesla system. Three main scan sequences were included: axial T2 weighted image (T2WI), diffusion weighted imaging (DWI), and dynamic contrast enhancement (DCE) images. Apparent diffusion coefficient (ADC) value was calculated by the highest b‐value (14). All mp-MRI images were evaluated by two radiologists with over 7 years of experience in prostate imaging. The index lesion was scored by PI‐RADS v2.1. The lesion volume (LV) was calculated via the following formula: horizontal plane diameter × sagittal plane diameter × coronal plane diameter × 0.523 (15).

### Statistical analysis

Continuous variables with non-normal distribution were expressed as median (interquartile range). Differences among groups were compared using the Mann–Whitney test. Categorical variables were presented as absolute and relative frequency, and were compared using chi-square test. Univariate and multivariate logistic regression analyses were conducted to identify independent risk factors for PCa or csPCa. Variables with statistical significance in the univariate logistic analysis were retained in the multivariate logistic analysis. Subsequently, the variables with statistical significance in the multivariate logistic analysis were included in the construction of the nomogram. Receiver operating curves (ROC) were established and the area under the curves (AUC) was calculated to assess the discriminatory ability of the models. Cutoff was found by the Youden’s index. Calibration curves were constructed to assess the extent of overestimation or underestimation of the models. Decision curves were made to determine the clinical net benefit. Internal validation was carried out with 1000 bootstrap resamples and Harrell’s C‐index was used to assess the discrimination performance. All analyses were performed with SPSS software (Version 27.0, IBM) and R software (version 3.6.2, R foundation for statistical computing, Vienna, Austria). Two-sided p < 0.05 was considered to indicate a statistical significance.

## Results

### Clinical characteristics and nomogram construction

**Figure 2:**
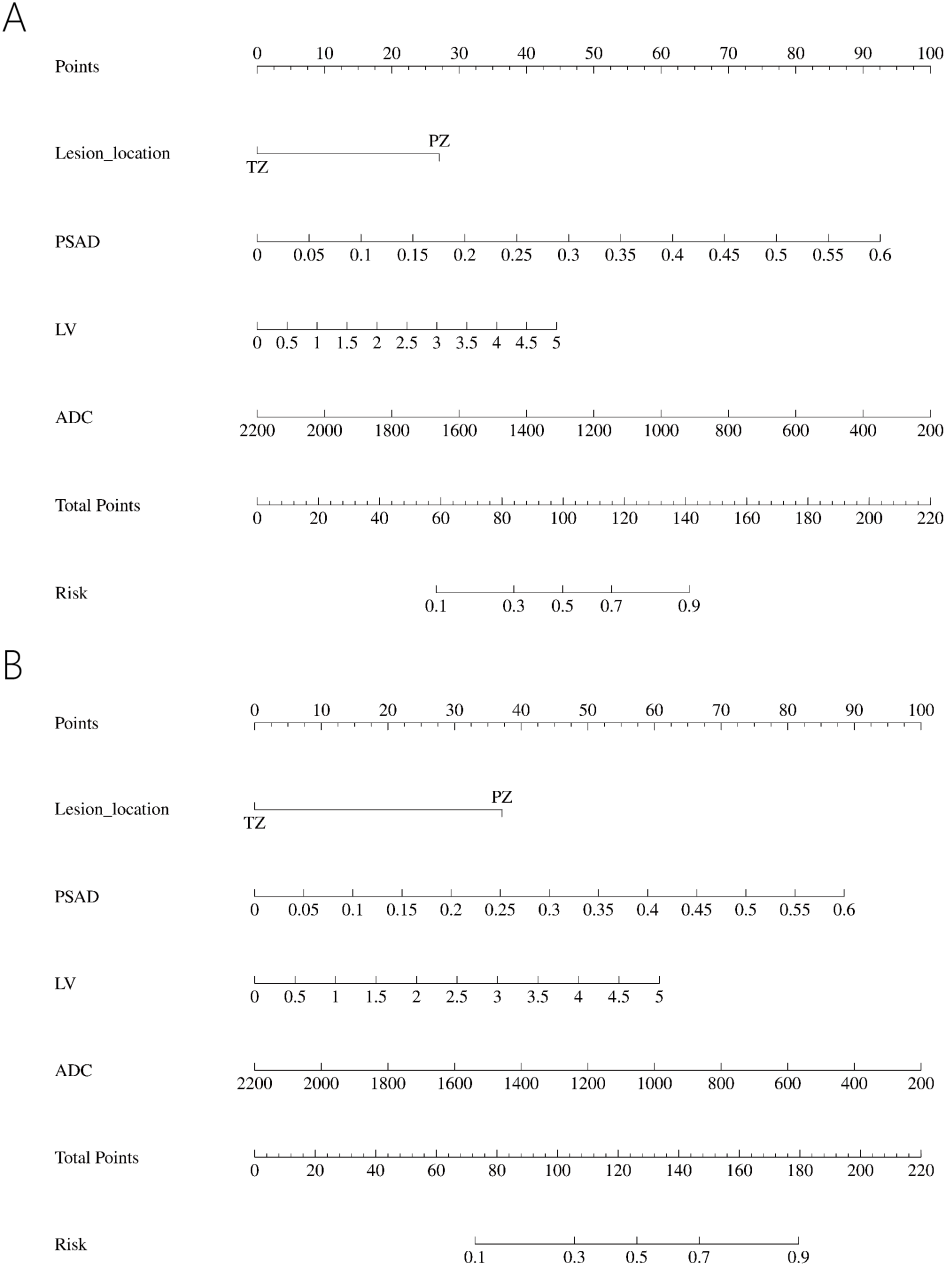
Nomogram predicting PCa **(A)** and predicting csPCa **(B)** in PI-RADS scores 1-3 population.

All the data including demographic, clinical and imaging features was presented in **Table 1**. A total of 327 patients were included in the study, of whom 224 (68.50%) were diagnosed with benign, 65 (19.87%) with csPCa, and 38 (11.62%) with cisPCa. Compared to those without PCa, lower f/t PSA, higher PSAD, smaller prostate volume (PV), larger LV, lower ADC, and lesions located mostly in the PZ were observed in patients with PCa or csPCa. Independent risk factors of PCa and csPCa were explored by univariable and multivariable logistic regression analyses (**Tables 2**, **3**). LV, ADC, PSAD and lesion location were screened out to establish the nomograms in patients with PI-RADS score ≤ 3 and PSA < 10 ng/ml (**Figures 2A, B**).

**Table 1:** Comparison of variables for benign and PCa, benign + cisPCa and csPCa in PI-RADS scores 1-3 population.

	PI-RADS scores 1-3			PI-RADS scores 1-3		
Variables	benign (n=224)	PCa (n=103)	*p*	benign + cisPCa (n=262)	csPCa (n=65)	*p*
**age, y** **(M, IQR)**	69 (64, 74)	69 (63, 73)	0.451	69 (63, 74)	70 (65, 75)	0.143
**tPSA, ng/ml** **(M, IQR)**	4.52 (2.64, 6.84)	6.02 (4.45, 8.10)	0.061	4.96 (2.78, 7.02)	6.02 (4.46, 8.13)	0.056
**fPSA, ng/ml** **(M, IQR)**	0.86 (0.51, 1.33)	0.87 (0.52, 1.28)	0.950	0.50 (0.22, 0.86)	0.54 (0.30, 0.87)	0.676
**f/t PSA** **(M, IQR)**	0.20 (0.16, 0.26)	0.16 (0.10, 0.21)	**<0.001**	0.20 (0.15, 0.2597)	0.16 (0.10, 0.22)	**0.002**
**PSAD, ng/ml^2^** **(M, IQR)**	0.07 (0.04, 0.11)	0.12 (0.08, 0.19)	**<0.001**	0.07 (0.04, 0.11)	0.14 (0.08, 0.20)	**<0.001**
**PV, ml** **(M, IQR)**	57.76 (40.31, 75.38)	41.75 (27.86, 58.68)	**<0.001**	55.45 (37.42, 74.38)	41.57 (27.20, 54.09)	**<0.001**
**LV, ml** **(M, IQR)**	0.25 (0.12, 0.58)	0.48 (0.24, 0.80)	**<0.001**	0.28 (0.12, 0.60)	0.52 (0.23, 0.86)	**<0.001**
**Lesion location, n**			**<0.001**			**<0.001**
**TZ**	206	67		236	37	
**PZ**	18	36		26	28	
**ADC, um^2^/s** **(M, IQR)**	1076.50 (984.50, 1181.00)	978.00 (852.00, 1100.00)	**<0.001**	1069.00 (973.50, 1175.25)	975.00 (843.50, 1129.50)	**<0.001**
**Gleason Score, n**			**-**			**-**
**<7**	0	38		38	0	
**7**	0	45		0	45	
**8**	0	8		0	8	
**9**	0	10		0	10	
**10**	0	2		0	2	

Boldface indicates statistically significant difference.

PCa, prostate cancer; cisPCa, clinically insignificant prostate cancer; csPCa, clinically significant prostate cancer; y, years old; SD, standard deviation; tPSA, total PSA; M, median; IQR, interquartile range; fPSA, free PSA; f/t PSA, free PSA divided by total PSA; PSAD, prostate‐specific antigen density; PV, prostate volume; LV, lesion volume; TZ, transitional zone; PZ, peripheral zone; ADC, apparent diffusion coefficient.

**Table 2:** Univariable and multivariable binary logistic regression analysis testing variables as independent risk factors of PCa in PI-RADS scores 1-3 population.

	Univariable analysis		Multivariable analysis	
Variables	Odds ratio (95% CI)	*p*	Odds ratio (95% CI)	*p*
**age**	0.98 (0.95, 1.01)	0.409	–	–
**tPSA**	1.15 (1.00, 1.26)	0.071	-	-
**fPSA**	0.88 (0.61, 1.28)	0.527	–	–
**f/tPSA**	0.001 (0.0001, 0.027)	**<0.001**	0.039 (0.001, 1.618)	0.088
**PSAD**	33560.17 (1017.54, 1106869.91)	**<0.001**	217.93 (3.03, 15671.42)	**0.014**
**PV**	0.97 (0.96, 0.98)	**<0.001**	0.99 (0.97, 1.00)	0.107
**LV**	1.51 (1.07, 2.13)	**0.016**	1.76 (1.18, 2.62)	**0.006**
**Lesion location**	0.16 (0.08, 0.30)	**<0.001**	0.25 (0.12, 0.54)	**<0.001**
**ADC**	0.996 (0.994, 0.998)	**<0.001**	0.997 (0.996, 0.999)	**0.005**

PCa, prostate cancer; tPSA, total PSA; fPSA, free PSA; f/t PSA, free PSA divided by total PSA, PSAD, prostate‐specific antigen density; PV, prostate volume; LV, lesion volume; ADC, apparent diffusion coefficient; CI, confidence interval. Boldface indicates statistically significant difference.

**Table 3:** Univariable and multivariable binary logistic regression analysis testing variables as independent risk factors of csPCa in PI-RADS scores 1-3 population.

	Univariable analysis		Multivariable analysis	
Variables	Odds ratio (95% CI)	*p*	Odds ratio (95% CI)	*p*
**age**	1.03 (0.99, 1.06)	0.093	–	–
**tPSA**	1.15 (1.00, 1.28)	0.077	-	-
**PSA**	1.02 (0.67, 1.55)	0.920	–	–
**f/tPSA**	0.01 (0.01, 0.36)	**0.011**	0.71 (0.01, 45.83)	0.874
**PSAD**	6241.05 (236.79,164494.63)	**<0.001**	117.01 (1.82, 7498.04)	**0.025**
**PV**	0.97 (0.96, 0.98)	**<0.001**	0.99 (0.97, 1.00)	0.176
**LV**	1.51 (1.07, 2.15)	**0.019**	1.77 (1.16, 2.70)	**0.008**
**Lesion location**	0.14 (0.07, 0.27)	**<0.001**	0.24 (0.11, 0.50)	**<0.001**
**ADC**	0.996 (0.994, 0.998)	**<0.001**	0.998 (0.996, 0.999)	**0.041**

csPCa, clinically significant prostate cancer; tPSA, total PSA; fPSA, free PSA; f/t PSA, free PSA divided by total PSA; PSAD, prostate‐specific antigen density; PV, prostate volume; LV, lesion volume; ADC, apparent diffusion coefficient; CI, confidence interval. Boldface indicates statistically significant difference.

To further explore the risk factors for PCa in patients with PI-RADS scores 1-2 and PI-RADS score 3. We divided the 327 patients into two subgroups: PI-RADS scores 1-2 and PI-RADS score 3. The demographic, clinical, and imaging features of the patients are listed in **Table 4**.

**Table 4:** Comparison of variables for benign and PCa in PI-RADS scores 1-2 population and PI-RADS score 3 population.

	PI-RADS scores 1-2			PI-RADS score 3		
Variables	Benign (n= 135)	PCa (n= 39)	*p*	Benign (n= 89)	PCa (n= 64)	*p*
**age, y** **(M, IQR)**	69 (65, 74)	70 (63, 76)	0.932	68 (63, 74)	69 (61, 72)	0.492
**tPSA, ng/ml** **(M, IQR)**	4.01 (2.19, 6.37)	4.76 (2.66, 7.49)	0.138	5.37 (3.59, 7.30)	6.02 (4.74, 8.14)	0.054
**fPSA, ng/ml** **(M, IQR)**	0.80 (0.41, 1.22)	0.66 (0.46, 1.03)	0.334	0.96 (0.63, 1.49)	1.04 (0.58, 1.39)	0.958
**f/t PSA** **(M, IQR)**	0.21 (0.17, 0.26)	0.14 (0.10, 0.22)	0.081	0.20 (0.13, 0.26)	0.16 (0.11, 0.21)	0.053
**PSAD, ng/ml^2^** **(M, IQR)**	0.06 (0.04, 0.10)	0.12 (0.07, 0.19)	**<0.001**	0.07 (0.05, 0.11)	0.13 (0.08, 0.19)	**<0.001**
**PV, ml** **(M, IQR)**	54.23 (34.02, 75.32)	39.14 (23.54, 53.81)	**<0.001**	59.28 (45.28, 75.43)	41.89 (31.13, 63.74)	**<0.001**
**LV, ml** **(M, IQR)**	0.23 (0.09, 0.51)	0.37 (0.18, 0.57)	0.093	0.30 (0.15, 0.74)	0.67 (0.26, 0.98)	0.073
**Lesion location, n**			**0.002**			**<0.001**
**TZ**	126	29		80	38	
**PZ**	9	10		9	26	
**ADC, um^2^/s** **(M, IQR)**	1104.00 (1008.00, 1200.00)	1060.00 (921.00, 1170.00)	**0.047**	1057.00 (972.00, 1159.00)	934.00 (842.25, 1031.50)	**<0.001**
**Gleason Score, n**			**-**			**-**
**<7**	0	13		0	25	
**7**	0	19		0	26	
**8**	0	2		0	6	
**9**	0	4		0	6	
**10**	0	1		0	1	

PCa, prostate cancer; y, years old; SD, standard deviation; tPSA, total PSA; M, median; IQR, interquartile range; fPSA, free PSA; f/t PSA, free PSA divided by total PSA; PSAD, prostate‐specific antigen density; PV, prostate volume; LV, lesion volume; TZ, transitional zone; PZ, peripheral zone; ADC, apparent diffusion coefficient. Boldface indicates statistically significant difference.

**Figure 3:**
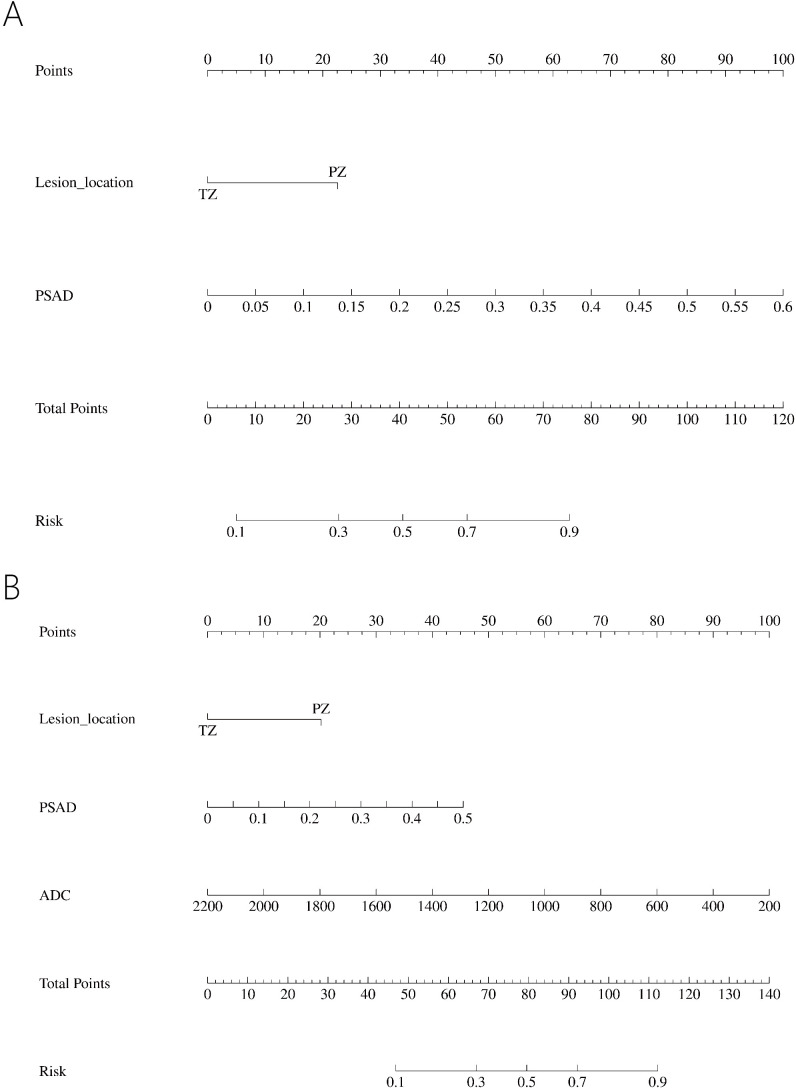
Nomogram predicting PCa in PI-RADS scores 1-2 population **(A)** and PI-RADS score 3 population **(B)**.

In the PI-RADS scores 1-2 subgroup, patients with PCa exhibited higher PSAD, lower PV, lower ADC, and more lesions in the PZ compared to those with benign. Independent risk factors for PCa were explored through univariable and multivariable logistic regression analyses (**Table 5**). PSAD and lesion location were screened out to develop the nomogram (**Figure 3A**).

**Table 5:** Univariable and multivariable binary logistic regression analysis testing variables as independent risk factors of PCa in PI-RADS scores 1-2 population.

	Univariable analysis		Multivariable analysis	
Variables	Odds ratio (95% CI)	*p*	Odds ratio (95% CI)	*p*
**age**	1.00 (0.95, 1.05)	0.846	–	–
**tPSA**	1.10 (0.96, 1.25)	0.143	-	-
**fPSA**	0.66 (0.35, 1.24)	0.201	–	–
**f/tPSA**	0.001 (0.001, 1.075)	0.079	-	-
**PSAD**	50351.76 (255.21, 9934017.37)	**<0.001**	3137.57 (11.45, 859763.51)	**0.005**
**PV**	0.97 (0.95, 0.98)	**0.001**	0.98 (0.96, 1.00)	0.067
**LV**	1.10 (0.58, 2.08)	0.752	–	–
**Lesion location**	0.20 (0.07, 0.55)	**0.002**	0.29 (0.09, 0.85)	**0.025**
**ADC**	0.997 (0.995, 0.999)	**0.029**	0.998 (0.996, 1.001)	0.306

PCa, prostate cancer; tPSA, total PSA; fPSA, free PSA; f/t PSA, free PSA divided by total PSA; PSAD, prostate‐specific antigen density; PV, prostate volume; LV, lesion volume; ADC, apparent diffusion coefficient; CI, confidence interval. Boldface indicates statistically significant difference.

In the PI-RADS score 3 subgroup, higher PSAD, lower PV, lower ADC, and lesions located mostly in the PZ were also observed in patients with PCa compared to those with benign. Logistic regression analysis identified PSAD, ADC, and lesion location as independent risk factors for PCa (**Table 6**), leading to the development of another nomogram for this subgroup (**Figure 3B**).

**Table 6:** Univariable and multivariable binary logistic regression analysis testing variables as independent risk factors of PCa in PI-RADS score 3 population.

	Univariable analysis		Multivariable analysis	
Variables	Odds ratio (95% CI)	*p*	Odds ratio (95% CI)	*p*
**age**	0.981 (0.94, 1.02)	0.395	–	–
**tPSA**	1.15 (0.91, 1.32)	0.052	-	-
**fPSA**	0.88 (0.53, 1.45)	0.621	–	–
**f/tPSA**	0.51 (0.01, 1.00)	0.056	-	-
**PSAD**	11536.43 (98.56, 1350239.85)	**<0.001**	397.89 (1.08, 146016.06)	**0.046**
**PV**	0.97 (0.96, 0.99)	**0.001**	0.99 (0.98, 1.01)	0.920
**LV**	1.55 (0.98, 2.45)	0.059	–	–
**Lesion location**	0.16 (0.07, 0.38)	**<0.001**	0.26 (0.09, 0.71)	**0.009**
**ADC**	0.995 (0.992, 0.997)	**<0.001**	0.996 (0.994, 0.999)	**0.007**

PCa, prostate cancer; tPSA, total PSA; fPSA, free PSA; f/t PSA, free PSA divided by total PSA; PSAD, prostate‐specific antigen density; PV, prostate volume; LV, lesion volume; ADC, apparent diffusion coefficient; CI, confidence interval. Boldface indicates statistically significant difference.

### Nomogram evaluation

All four nomograms showed acceptable discrimination through internal validation with 1000 bootstrap resamples. In patients with PI-RADS scores 1-3, the C-index was 0.775 for PCa and 0.779 for csPCa. In patients with PI-RADS scores 1-2, the C-index was 0.749 for PCa. In patients with PI-RADS score 3, the C-index was 0.777 for PCa.

**Figure 4:**
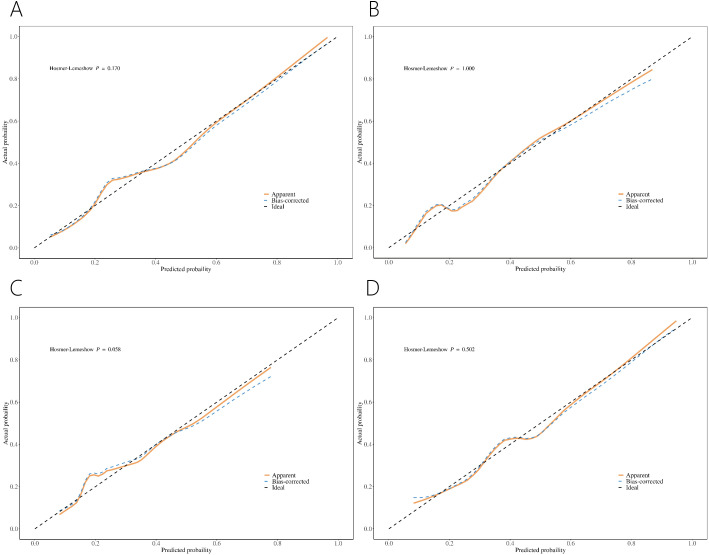
Calibration curve of nomograms for PCa **(A)** and csPCa **(B)** in PI-RADS scores 1-3 population. Calibration curve of nomograms for PCa in PI-RADS scores 1-2 population **(C)** and PI-RADS score 3 population **(D)**.

Calibration curves showed good concordance between the predicted probability and the actual probability for the four nomograms. For patients with PI-RADS scores 1-3, the risk may be overestimated when the threshold exceeds 36% for PCa and 56% for csPCa (**Figures 4A, B**). For patients with PI-RADS scores 1-2, the risk may be overestimated when the threshold is above 33% for PCa (**Figure 4C**). For patients with PI-RADS score 3, the risk may be overestimated when the threshold is greater than 41% for PCa (**Figure 4D**).

**Figure 5:**
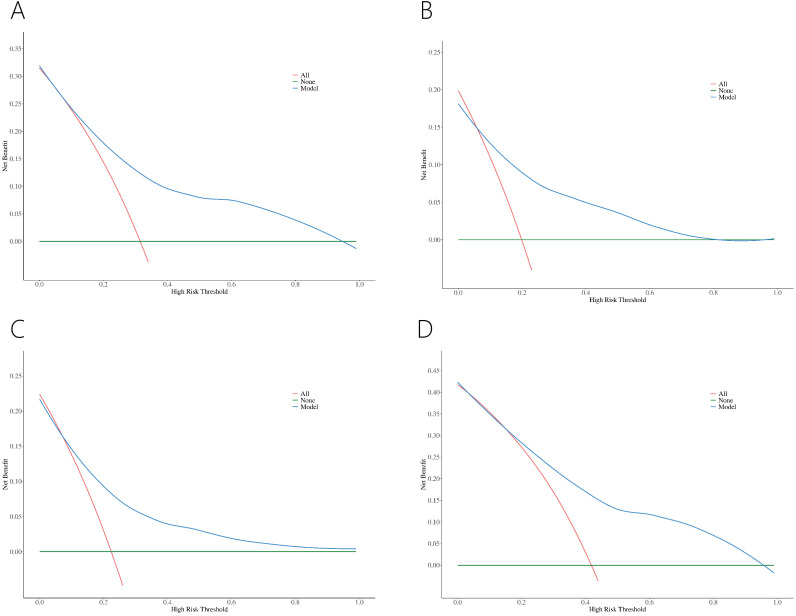
DCA of nomograms for PCa **(A)** and csPCa **(B)** in PI-RADS scores 1-3 population. DCA of nomograms for PCa in PI-RADS scores 1-2 population **(C)** and PI-RADS score 3 population **(D)**.

Decision curve analysis (DCA) revealed greater net benefits for predicted probabilities between 10% and 95% for the four nomograms (**Figure 5**).

**Figure 6:**
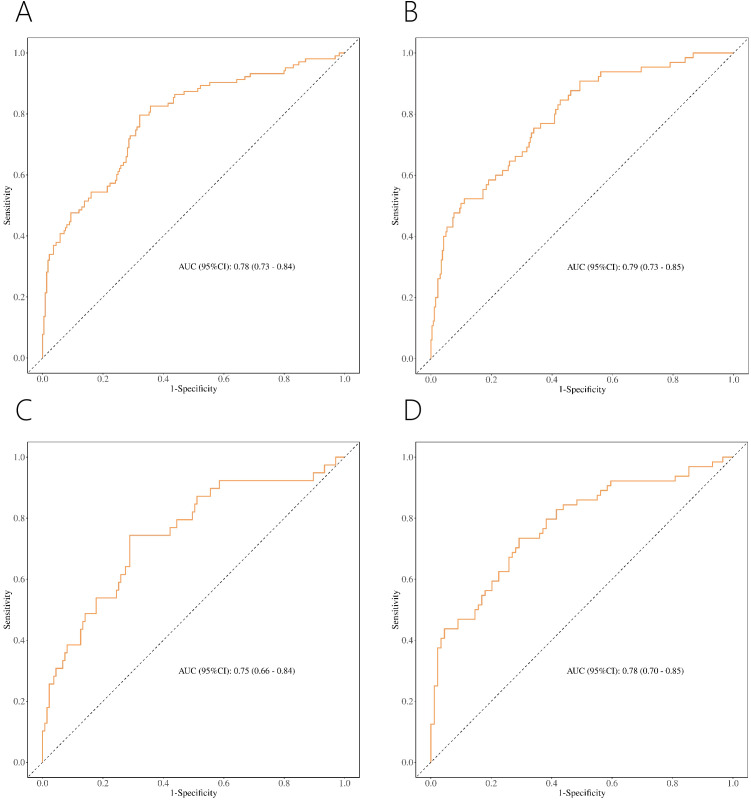
ROC of nomograms for PCa **(A)** and csPCa **(B)** in PI-RADS scores 1-3 population. ROC of nomograms for PCa in PI-RADS scores 1-2 population **(C)** and PI-RADS score 3 population **(D)**.

ROC showed acceptable discrimination for the four nomograms (**Figure 6**, **Table 7**). For patients with PI-RADS scores 1-3, the AUC for predicting PCa and csPCa were 0.78 and 0.79, with sensitivity of 0.68 and 0.57, and specificity of 0.80 and 0.83, respectively (**Figures 6A, B**). For patients with PI-RADS scores 1-2, the AUC for predicting PCa was 0.75, with a sensitivity of 0.71 and specificity of 0.74 (**Figure 6C**). For patients with PI-RADS score 3, the AUC for predicting PCa was 0.78, with a sensitivity of 0.71 and specificity of 0.72 (**Figure 6D**). Thus, dividing the samples into PI-RADS scores 1-2 subgroup and PI-RADS score 3 subgroups did not improve the AUC of predicting PCa.

**Table 7:** Nomograms’ discrimination for PCa or csPCa.

	AUC (95% CI)	Cutoff *	Sensitivity	Specificity	Youden’s index	PPV	NPV
**nomogram for PCa in PI-RADS scores 1-3 population**	0.78 (0.73-0.84)	0.25	0.68	0.80	0.48	0.88	0.53
**nomogram for csPCa in PI-RADS scores 1-3 population**	0.79 (0.73-0.85)	0.12	0.57	0.83	0.40	0.93	0.32
**nomogram for PCa in PI-RADS scores 1-2 population**	0.75 (0.66-0.84)	0.19	0.71	0.74	0.45	0.91	0.43
**nomogram for PCa in PI-RADS score 3 population**	0.78 (0.70-0.85)	0.35	0.71	0.72	0.43	0.78	0.64

PCa, prostate cancer; csPCa, clinically significant prostate cancer; AUC, area under the receiver operating curves; CI, confidence interval; NPV, negative predictive value; PPV, positive predictive value.

*Cutoff with best diagnostic performance.

The number of avoided biopsies was calculated at a sensitivity of 0.85 to further analyze the clinical utility of each nomogram (**Table 8**). Biopsy could be avoided by at least 36.60% through nomograms.

**Table 8:** Avoiding biopsy numbers at a sensitivity of 0.85 for nomogram.

	Sensitivity	Specificity	Cutoff	Biopsies avoided, n (%)	Missed cases, n (%)
**nomogram for PCa in PI-RADS scores 1-3 population**	0.85	0.57	0.21	143 (43.73)	15 (14.56)
**nomogram for csPCa in PI-RADS scores 1-3 population**	0.85	0.55	0.12	154 (47.09)	10 (15.38)
**nomogram for PCa in PI-RADS scores 1-2 population**	0.85	0.50	0.13	74 (42.52)	6 (15.38)
**nomogram for PCa in PI-RADS score 3 population**	0.85	0.52	0.30	56 (36.60)	10 (15.62)

PCa, prostate cancer; csPCa, clinically significant prostate cancer.

## Discussion

Multiple models have been developed to predict PCa and csPCa, focusing primarily on population with PI-RADS score 3 or PSA < 10 ng/ml, but fewer studies have addressed the population with PI-RADS scores 1-3, regardless of PSA level (11, 16, 17). To our knowledge, no model has been developed to predict PCa or csPCa in special population of PI-RADS scores 1-3 and PSA < 10 ng/ml. However, PCa and csPCa cases do occur in the PI-RADS scores 1-3 and PSA < 10 ng/ml, and are challenging to detect (18, 19). Accordingly, how to identify PCa or csPCa in the special population is still a challenge. In this study, we aimed to address this interesting issue.

We found that PSAD, LV, lesion location and ADC are independent risk factors for PCa. These findings are consistent with other studies highlighting these factors as predictors for PCa and csPCa in PI-RADS score 3 (20–23). Typically, lower ADC, higher PSAD, larger LV, and lesions in the PZ increase the likelihood of PCa or csPCa (20–24). Although some studies suggest age may also be a risk factor, we did not include it in our analysis due to the lack of significant age differences in our cohort and the conflicting results reported in the literature (25, 26).

We developed nomograms with acceptable discriminability for predicting PCa and csPCa in patients with PI-RADS scores 1-3, achieving AUC of 0.78 and 0.79, respectively (**Figures 6A, B**). Given the different risks of PCa for PI-RADS scores 1-2 versus PI-RADS score 3, and the varying recommendations for PBx (PBx is generally not recommended for PI-RADS scores 1-2 and is controversial for PI-RADS score 3) (9, 17), we further divided the population into two subgroups (PI-RADS scores 1-2 and PI-RADS score 3 subgroups). This allowed us to explore whether PCa could be predicted more accurately. Although the AUC for predicting PCa was 0.75 in PI-RADS scores 1-2 and 0.78 in PI-RADS score 3 (**Figures 6C, D**), these did not show improved discriminative ability compared to the nomogram for the PI-RADS scores 1-3 (AUC = 0.78). Accordingly, the nomogram for PI-RADS scores 1-3 appears to be the more practical choice for clinical application.

Previous studies have developed predictive models for PCa or csPCa, specifically for those with PI-RADS score 3 (15, 27–29). For example, Hectors et al. (27) developed radiomics classifier with an AUC of 0.76 for predicting csPCa in PI-RADS score 3, while Martorana et al. (15) developed nomogram with an AUC of 0.86 for the same score. These models demonstrate good discriminability. However, these studies did not address prediction in the context of PSA levels in the “gray zone” (PSA < 10 ng/ml). Research has indicated that PCa may be missed in the gray zone, potentially leading to delayed diagnosis and missed treatment opportunities (16). Our study, focusing on patients with PSA < 10 ng/ml and PI-RADS score 3, achieved an AUC of 0.78 for predicting PCa (**Figure 6D**), which underscores the importance of our findings for this specific population.

Few studies have developed nomograms for PCa or csPCa specifically in populations with PI-RADS scores 1-3 (12, 17). For example, Zhang et al. (12) developed nomogram to predict the absence of PCa and csPCa, achieving AUC of 0.75 and 0.76, respectively. However, their model may not be as clinically applicable because it does not directly provide a probability estimate for PCa or csPCa (30). Additionally, their study did not specifically address patients with PSA levels in the gray zone. In contrast, our nomogram, designed for PI-RADS scores 1-3 and PSA < 10 ng/ml, may be better suited for clinical practice (**Figures 6A, B**).

To our knowledge, no nomograms have been reported for predicting PCa in the population with PI-RADS scores 1-2 and PSA < 10 ng/ml (31). Although studies by Abdul et al. (17) and Buisset et al. (32) identified the presence of PCa in individuals with PI-RADS scores of 1-2, they did not develop prediction models. We constructed a nomogram (AUC = 0.75) specifically for this population, which may offer a valuable clinical tool for predicting PCa in individuals with PI-RADS scores 1-2 and PSA < 10 ng/ml (**Figure 6C**).

Our models demonstrated good concordance between predicted and actual probabilities (**Figure 4**). However, in clinical practice, it is important to evaluate whether the risk of PCa is being overestimated or underestimated. For instance, in a population with PI-RADS scores 1-3 and PSA < 10 ng/ml, the nomogram may overestimate the risk of PCa when the predicted probability exceeds 36%. Further evaluation is necessary to decide whether to proceed with PBx.

At a sensitivity of 85%, biopsy could be avoided by at least 36.60% through nomograms (**Table 8**). Although the results differed slightly from those of Hu et al. (11) and Yang et al. (22), our nomograms still had acceptable clinical utility.

This study has several limitations. First, it was conducted at a single center and lacked external validation, although internal validation through bootstrap demonstrated good discriminability. Second, the retrospective nature of the study introduces the potential of selection bias. Finally, the relatively small sample size limited further exploration of models for csPCa in patients with PI-RADS scores 1-2 and PI-RADS score 3.

In conclusion, for the specific population with PI-RADS scores 1-3 and PSA < 10 ng/ml, research on detecting PCa and csPCa to avoid unnecessary PBx is limited. To address this gap, we developed novel nomograms with acceptable discriminability for predicting PCa and csPCa in this population. These models can assist urologists in making informed decisions about whether to proceed with a PBx.

## Data Availability

The original contributions presented in the study are included in the article/supplementary material. Further inquiries can be directed to the corresponding author.
